# Felt Ambivalence Weakens the Attitude–Intention Pathway for Regular Leisure-Time Physical Activity in Chinese Adolescents: A Three-Wave Prospective Study

**DOI:** 10.3390/bs16040545

**Published:** 2026-04-06

**Authors:** Yaogang Han, Yubing Wang, Pan Li, Guohua Zheng

**Affiliations:** 1 School of Physical Education, Shanghai University of Sport, Shanghai 200438, China; hanyaogang@sus.edu.cn; 2 Department of Human Movement Studies & Special Education, Old Dominion University, Norfolk, VA 23529, USA; y8wang@odu.edu; 3 School of Athletic Performance, Shanghai University of Sport, Shanghai 200438, China; lipan@sus.edu.cn; 4 School of Journalism and Communication, Shanghai University of Sport, Shanghai 200438, China

**Keywords:** adolescents, leisure-time physical activity, felt ambivalence, attitude strength, intention formation

## Abstract

**Background**: Favorable attitudes toward regular leisure-time physical activity may not always translate into intention if adolescents feel ambivalent about the behavior. This study tested whether felt ambivalence weakens the prospective attitude–intention association and the indirect effect of attitude on later behavior through intention. **Methods**: Chinese adolescents (N = 1714; Grades 7–12; mean age = 15.0 years) completed a three-wave survey at approximately two-week intervals. Wave 1 assessed attitudes toward regular leisure-time moderate-to-vigorous physical activity, felt ambivalence, and physical activity habit; Wave 2 assessed intention; and Wave 3 assessed leisure-time physical activity. Moderated mediation was tested in a structural equation model adjusting for habit, gender, and grade. **Results**: More favorable baseline attitudes predicted stronger intention two weeks later, and intention predicted greater self-reported leisure-time physical activity at follow-up. Felt ambivalence significantly moderated the attitude–intention pathway such that the association was weaker at higher levels of ambivalence. The conditional indirect effect of attitude on later leisure-time physical activity through intention was significant at low, mean, and high ambivalence, but decreased as ambivalence increased. **Conclusions**: Favorable attitudes may be insufficient when adolescents remain conflicted about physical activity. The present study provides prospective support for a theoretically relevant moderation pattern in which felt ambivalence weakens the attitude–intention pathway, but it does not establish ambivalence as a key explanatory mechanism.

## 1. Introduction

Regular physical activity (PA) during adolescence is associated with multiple physical and mental health benefits, yet most adolescents worldwide do not achieve recommended activity levels (Guthold et al., 2020; van Sluijs et al., 2021). Reviews also point to continuing gaps for older adolescents and non-Western contexts (van Sluijs et al., 2021). Leisure-time moderate-to-vigorous physical activity (MVPA) deserves particular attention because it is more discretionary than compulsory school activity and therefore depends heavily on adolescents’ own evaluations and decisions. Understanding how adolescents form intentions for regular leisure-time MVPA is therefore theoretically and practically important.

The reasoned action approach (RAA) and the theory of planned behavior (TPB) provide a useful starting point. These models propose that attitudes toward a behavior are key antecedents of intention, and that intention is the most proximal motivational predictor of later behavior (Ajzen, 1991; Fishbein & Ajzen, 2010). This general pattern is already well established in health-behavior and physical-activity research (Hagger et al., 2002; R. R. C. McEachan et al., 2011) and continues to inform adolescent leisure-time PA studies (Pasi et al., 2021; Su et al., 2024). The less examined question is whether equally favorable attitudes differ in strength—and therefore differ in how effectively they generate intention.

A positive attitude is not necessarily a strong one. Attitude research distinguishes between attitude valence—how favorable or unfavorable an evaluation is—and attitude strength, or the extent to which an attitude is durable and impactful (Petty et al., 2023). Accordingly, the more specific question is why some adolescents who evaluate regular leisure-time MVPA positively still fail to form strong intentions. Rather than positioning the study as a departure from intention–behavior gap research, the present study refines reasoned action/TPB models by testing whether differences in attitude strength help explain why equally favorable attitudes do not translate equally into intention. This directs attention to felt ambivalence as a plausible factor that may weaken attitude strength.

Felt ambivalence refers to the subjective experience of being mixed, conflicted, or undecided about an attitude object (Priester & Petty, 1996). In the context of leisure-time MVPA, adolescents may simultaneously view regular activity as beneficial, valuable, or enjoyable while also experiencing countervailing reactions—for example, that it is effortful, time-consuming, socially uncomfortable, or in tension with academic demands and other leisure alternatives. Under such conditions, an overall attitude score can remain favorable, yet the attitude may be less settled and therefore less able to generate commitment. Consistent with this view, ambivalence has long been treated as a strength-related attitude attribute: less ambivalent attitudes are more predictive of subsequent intentions and behavior and more resistant to persuasion than highly ambivalent attitudes (Armitage & Conner, 2000; Petty et al., 2023). Within theory of planned behavior research, ambivalence has also been shown to alter the strength of prospective model relations in health-behavior contexts (Conner et al., 2003).

Despite its relevance, this attitude-strength perspective has received limited attention in adolescent physical activity research. Recent work on adolescents’ leisure-time PA has more often extended TPB models with additional motivational constructs than examined whether the attitude component itself is strong enough to produce intention (Pasi et al., 2021; Su et al., 2024). Thus, the specific gap addressed here is not whether attitudes and intentions matter in adolescent PA—that issue is already well established—but whether felt ambivalence qualifies the prospective attitude–intention association within this literature. To our knowledge, direct evidence on ambivalence in adolescent PA remains limited. The closest direct PA evidence we identified comes from adolescents with critical congenital heart disease, among whom greater ambivalence toward physical activity was associated with lower objectively measured MVPA and poorer cardiorespiratory fitness (Fox et al., 2023). That study is important, but it leaves open whether felt ambivalence operates similarly in a large general adolescent sample and whether it specifically weakens the prospective pathway from attitude to intention for regular leisure-time MVPA. Accordingly, the distinct contribution of the present study is not to introduce ambivalence or attitude strength as new concepts, but to test prospectively whether felt ambivalence functions as a boundary condition on the attitude–intention pathway for regular leisure-time MVPA in a general adolescent school sample, while accounting for PA habit as a rival process.

A further reason to isolate this mechanism is that adolescent physical activity is not governed exclusively by reflective decision making. Habits can develop through repeated activity in stable contexts and may come to be experienced as low effort, automatic, and relatively independent of goals and intentions (Hagger, 2019). More broadly, meta-analytic work on reasoned action models shows that including past behavior attenuates model effects, consistent with the possibility that nonconscious processes partly underlie repeated health behaviors (Hagger et al., 2018). For the present purpose, however, habit is best treated as a rival process that should be controlled, rather than as a coequal theoretical focus. Doing so permits a cleaner test of whether felt ambivalence weakens the extent to which favorable attitudes are translated into intentions.

The present study tested this question in a three-wave prospective design among a large general school sample of Chinese adolescents. Integrating reasoned action logic with attitude-strength theory, we examined whether more favorable baseline attitudes toward regular leisure-time MVPA predicted stronger subsequent intentions, whether this attitude–intention relationship was weaker among adolescents reporting greater felt ambivalence, and whether intention in turn predicted later leisure-time physical activity. Accordingly, rather than replacing established intention–behavior accounts, but to refine them by testing whether felt ambivalence serves as a theoretically meaningful first-stage moderator of intention formation. We further tested whether the indirect effect of baseline attitude on later leisure-time physical activity through intention was conditional on felt ambivalence. We hypothesized that more positive attitudes would predict stronger subsequent intention, that felt ambivalence would weaken the positive attitude–intention association, and that the indirect effect of attitude on later leisure-time physical activity through intention would therefore be weaker at higher levels of felt ambivalence.

## 2. Method

### 2.1. Participants and Design

This study used a three-wave prospective design with approximately two-week intervals between adjacent waves. A relatively short lag was chosen to capture a proximal attitude → intention → behavior sequence, consistent with reasoned action/TPB work indicating that intention–behavior correspondence is typically strongest when intention is assessed close in time to behavior and tends to weaken as the interval increases (Fishbein & Ajzen, 2010; Randall & Wolff, 1994). The spacing was also compatible with repeated school-based administration across coordinated data-collection sessions. Wave 1 assessed students’ attitudes toward regular leisure-time moderate-to-vigorous physical activity (MVPA), felt ambivalence toward that behavior, and PA habit strength. Wave 2 assessed intention to perform regular leisure-time MVPA during the next month. Wave 3 assessed the behavioral outcome. Participants were recruited from six middle schools and four high schools in Shanghai, China, through school access facilitated by the researchers’ professional networks. The study protocol was reviewed and approved by the Shanghai University of Sport Institutional Review Board. Because participants were adolescents, written informed consent was obtained from parents or legal guardians, and written assent was obtained from students before data collection. Participation was voluntary, and responses were collected anonymously. Of the 1844 students who completed Wave 1, 1809 (98.1%) completed Wave 2, and 1714 (94.7% of Wave 2; 93.0% of Wave 1) completed Wave 3. The final analytic sample comprised these 1714 students with complete data on all focal variables and covariates. Participants were enrolled in Grades 7 through 12, including Grade 7 (n = 335, 19.5%), Grade 8 (n = 299, 17.4%), Grade 9 (n = 259, 15.1%), Grade 10 (n = 287, 16.7%), Grade 11 (n = 288, 16.8%), and Grade 12 (n = 246, 14.4%). The sample included 859 boys (50.1%) and 855 girls (49.9%). The mean age of the analytic sample was 15.0 years (SD = 1.7).

### 2.2. Variables and Measures

#### 2.2.1. Physical Activity Attitude

Wave 1 attitude toward regular leisure-time MVPA was assessed with six bipolar semantic differential items. Three items represented affective or experiential evaluation (e.g., interesting, relaxing, exciting), and three represented cognitive or instrumental evaluation (e.g., valuable, beneficial, wise). This measurement strategy is consistent with Theory of Planned Behavior and Reasoned Action questionnaire recommendations and with work distinguishing affective/experiential and cognitive/instrumental components of attitude (Francis et al., 2004; R. McEachan et al., 2016). Higher scores reflected more favorable evaluations of regular leisure-time MVPA. Because the affective and cognitive subdimension scores were highly correlated in the present sample (r = 0.81), the six items were averaged to form a single overall attitude composite.

#### 2.2.2. Physical Activity Habit

Wave 1 PA habit strength was measured using the four-item automaticity subscale of the Self-Report Habit Index (Gardner et al., 2012; Verplanken & Orbell, 2003). These items emphasize automaticity and minimal deliberation in activity performance, such as doing PA automatically, without conscious thought, or beginning the behavior before fully realizing one has started. This item content is closely aligned with the automaticity core of the Self-Report Habit Index tradition (Verplanken & Orbell, 2003). Items were averaged so that higher values indicated stronger PA habit.

#### 2.2.3. Felt Ambivalence Toward Physical Activity

Wave 1 felt ambivalence toward regular leisure-time MVPA was measured with three direct items assessing the extent to which students experienced difficulty deciding, mixed positive and negative views, and conflicting views when expressing their overall attitude toward the behavior. This direct approach captures subjective or felt ambivalence rather than inferring ambivalence indirectly from separate positive and negative evaluations (Priester & Petty, 1996). Prior theory of planned behavior research has shown that attitudinal ambivalence can weaken the predictive strength of attitudes in prospective models (Conner et al., 2003). Items were averaged so that higher values indicated greater felt ambivalence.

#### 2.2.4. Physical Activity Intention

Wave 2 intention was assessed with three items measuring intention, plan, and perceived likelihood of engaging in regular leisure-time MVPA during the next month. In the questionnaire, the target behavior was explicitly defined as doing MVPA in leisure time at least three times per week for at least 20 min each time. This direct specification of action, context, and time frame follows standard TPB/RAA measurement principles (Francis et al., 2004). The three items were averaged so that higher values indicated stronger intention.

#### 2.2.5. Leisure-Time Physical Activity

The Wave 3 behavioral outcome was a two-item leisure-time PA index. One item assessed how many days per week students typically engaged in leisure-time MVPA, and the second assessed the regularity with which they engaged in leisure-time MVPA. Both items were coded so that higher values indicated more frequent and more regular participation, and the mean of the two items was used as a continuous index of leisure-time PA. This outcome was used because it corresponded most closely to the focal attitude and intention measures in action, context, and behavioral specificity. In reasoned action research, stronger correspondence between intention and behavior measures generally improves the interpretability of prospective intention–behavior relations (Francis et al., 2004; R. McEachan et al., 2016). This measure should be viewed as a brief behavioral indicator rather than a comprehensive psychometric assessment of adolescent PA. It was selected to reduce respondent burden in repeated school-based administration, but its brevity necessarily limits content coverage and does not fully capture the multidimensional nature of leisure-time MVPA (e.g., duration, intensity variability, or activity type).

### 2.3. Data Collection

Data were collected in-person during school-based group administration sessions coordinated with PE teachers and school staff. Researchers were present throughout data collection to answer students’ questions and to standardize administration procedures. At Wave 1, students completed the baseline questionnaire assessing PA attitude, PA ambivalence, and PA habit strength. Approximately two weeks later, students completed the Wave 2 questionnaire assessing intention to engage in regular leisure-time MVPA during the following month. Approximately two weeks after Wave 2, students completed the Wave 3 questionnaire assessing leisure-time PA behavior.

### 2.4. Data Analysis

All analyses were conducted in R (version 4.5.0). Descriptive statistics, internal consistency estimates, and zero-order Pearson correlations were computed first. The primary hypotheses were then tested using an observed-variable moderated mediation path model estimated within a structural equation modeling (SEM) framework in the lavaan package for R (Rosseel, 2012). Observed composite scores were used rather than latent interaction models to retain parsimony and because the focal scales demonstrated high internal consistency. Wave 1 PA attitude and Wave 1 ambivalence were mean-centered, and their product term was used to test whether felt ambivalence moderated the Wave 1 attitude → Wave 2 intention pathway. The model specified Wave 2 intention as the mediator linking Wave 1 attitude to Wave 3 leisure-time PA, with Wave 1 ambivalence moderating the first stage of that indirect effect. Participants were nested within 10 schools. Because the present study was not designed to estimate school-level effects and the number of schools was limited, we retained an individual-level SEM rather than fitting a multilevel model. The implications of this decision for statistical inference are acknowledged in the limitations section. Primary analyses were conducted on the complete-case sample because the focal moderated mediation model required temporally ordered data across all three waves. Given the high retention across waves, this approach resulted in limited case loss. To evaluate potential attrition bias, participants retained in the analytic sample were compared with those not retained on available Wave 1 focal constructs (PA attitude, PA habit, and felt ambivalence). PA habit, gender, and grade were included as covariates of both Wave 2 intention and Wave 3 leisure-time PA.

Indirect effects were evaluated using 5000 bootstrap resamples and percentile-based confidence intervals. Moderated mediation was evaluated using the index of moderated mediation (Hayes, 2015). Model fit was evaluated using the chi-square test, comparative fit index (CFI), root mean square error of approximation (RMSEA), and standardized root mean square residual (SRMR). Because the final model had only one degree of freedom, fit indices were interpreted cautiously and alongside the substantive parameter estimates.

## 3. Results

Of the 1844 students assessed at Wave 1, 1809 (98.1%) completed Wave 2 and 1714 (94.7% of Wave 2; 93.0% of Wave 1) completed Wave 3. Independent-samples tests showed no significant baseline differences between the complete-case sample and those not retained on Wave 1 PA attitude, PA habit, or felt ambivalence (*p* = 0.393 to 0.951), suggesting limited evidence of attrition bias on the focal baseline constructs. Descriptive statistics, internal consistency estimates, and zero-order correlations are presented in Table 1. Internal consistency was high for the focal composites (αs = 0.90–0.95), and the zero-order associations were directionally consistent with the hypothesized model.

### 3.1. The Moderated Mediation Model

**Figure 1:**
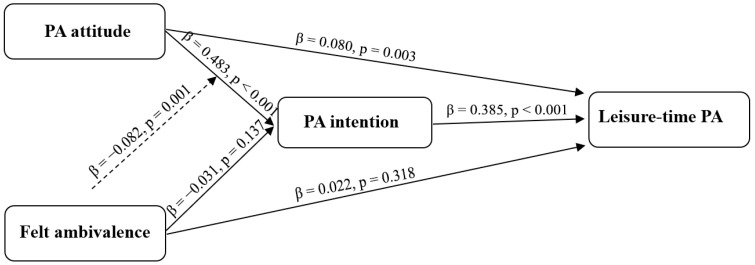
Moderated mediation model linking Wave 1 PA attitude and felt ambivalence to Wave 3 leisure-time PA via Wave 2 PA intention. Standardized coefficients (β) and *p* values are shown. Solid lines indicate direct paths, and the dashed line indicates the interaction effect testing moderation by felt ambivalence.

The moderated mediation model is shown in Figure 1, and the corresponding parameter estimates are presented in Table 2. Because the final model had only one degree of freedom, the global fit statistics are reported for completeness but should be interpreted cautiously. In this context, the substantive interpretation rests primarily on the parameter estimates and explained variance rather than on the fit indices alone, although the observed values (χ^2^ = 0.001, *p* = 0.972, CFI = 1.00, RMSEA = 0.000, SRMR < 0.001) did not indicate obvious model misfit. The model explained 46.8% of the variance in Wave 2 intention and 35.0% of the variance in Wave 3 leisure-time PA. Wave 1 attitude toward regular leisure-time MVPA positively predicted Wave 2 intention, b = 0.577, SE = 0.031, z = 18.54, *p* < 0.001, β = 0.483 (Table 2; Figure 1). Thus, adolescents who evaluated regular leisure-time MVPA more positively at baseline reported stronger intentions two weeks later.

### 3.2. Moderating Role of Felt Ambivalence

The main effect of Wave 1 ambivalence on Wave 2 intention was not statistically significant, b = −0.024, SE = 0.016, z = −1.49, *p* = 0.137, β = −0.031. However, the interaction between Wave 1 attitude and Wave 1 ambivalence was significant and negative, b = −0.058, SE = 0.017, z = −3.41, *p* = 0.001, β = −0.082 (Table 2). This pattern indicates that ambivalence did not simply lower intention in a uniform way. Rather, it weakened the extent to which positive attitudes translated into later intention.

**Figure 2:**
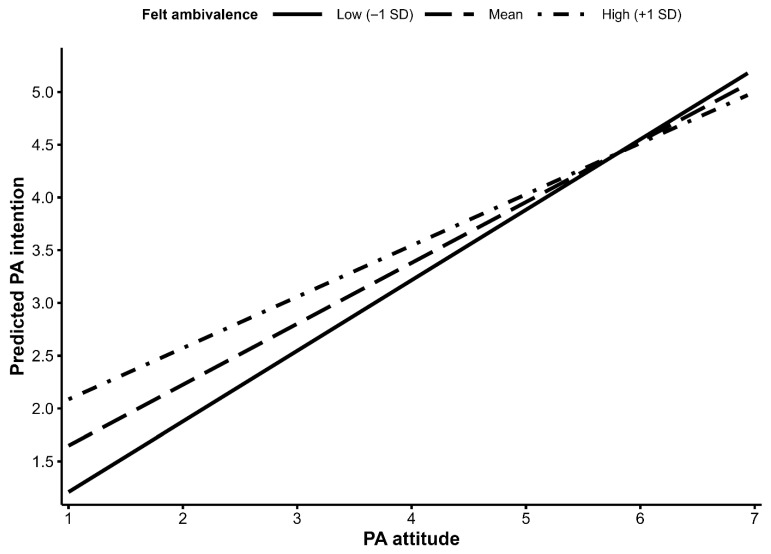
Simple slopes of the association between Wave 1 PA attitude and Wave 2 PA intention at low, mean, and high levels of felt ambivalence. Lines represent model-implied predicted Wave 2 PA intention across the original Wave 1 PA attitude scale at low (−1 SD), mean, and high (+1 SD) levels of felt ambivalence. More positive attitudes were associated with stronger intention at all three levels of ambivalence, but the attitude–intention association was weaker at higher levels of ambivalence. Predicted values were derived from the adjusted model including PA habit, gender, and grade, with covariates held constant in the plotted predictions.

As shown in Figure 2, the simple slopes remained positive across low, mean, and high levels of ambivalence, but the attitude–intention association became progressively weaker as ambivalence increased. Although the standardized interaction coefficient was modest (β = −0.082), the simple-slope pattern indicates a meaningful attenuation in the original metric of the model. Using the estimated coefficients and ±1 SD values of ambivalence, the attitude → intention slope was approximately 0.67 at low ambivalence, 0.58 at the mean, and 0.49 at high ambivalence. Thus, across the low-to-high ambivalence range, the predictive strength of attitude for later intention was reduced by about 27%. In substantive terms, positive attitudes predicted stronger subsequent intention at all levels of ambivalence, but they were less effective in generating intention among adolescents who also felt more conflicted about the behavior.

### 3.3. Prediction of Wave 3 Leisure-Time Physical Activity

Wave 2 intention positively predicted Wave 3 leisure-time PA, β = 0.385, and Wave 1 attitude retained a smaller direct association with Wave 3 leisure-time PA, β = 0.080, indicating partial rather than full mediation (Table 2; Figure 1). By contrast, the direct effect of Wave 1 ambivalence on Wave 3 leisure-time PA was not significant. Among the covariates, Wave 1 habit positively predicted both intention and later leisure-time PA; girls reported lower intention and leisure-time PA than boys, and higher grade was associated with lower leisure-time PA but not intention.

### 3.4. Conditional Indirect Effects

Bootstrap analyses supported the hypothesized moderated mediation (Table 2). The indirect effect of Wave 1 attitude on Wave 3 leisure-time PA through Wave 2 intention remained positive across low, mean, and high ambivalence, but decreased as ambivalence increased. From low to high ambivalence, the indirect effect declined from 0.268 to 0.194 (about 28%), and the index of moderated mediation was negative, b = −0.023, 95% CI [−0.039, −0.011]. Thus, the positive indirect effect of attitude on later leisure-time PA through intention became significantly weaker as felt ambivalence increased. Taken together, these findings are consistent with an attitude-strength account of adolescent leisure-time PA, but they also indicate that the moderation is modest in standardized terms and substantively meaningful mainly as a partial attenuation of intention formation rather than as a large effect.

## 4. Discussion

The present study examined whether favorable attitudes toward regular leisure-time MVPA may fail to crystallize into strong intentions among adolescents. The findings are generally consistent with that central premise. More positive baseline attitudes predicted stronger intention, and stronger intention predicted greater self-reported leisure-time physical activity. At the same time, the evidence regarding ambivalence should be interpreted cautiously. The moderation was statistically reliable but modest, and its clearest implication concerns short-term intention formation rather than a broad or deterministic effect on later behavior. Because the behavioral outcome was self-reported, however, these behavior-related associations should be interpreted cautiously, especially in terms of their magnitude. Notably, ambivalence did not emerge as a uniformly negative predictor of intention or later PA. Instead, it was associated with variation in how strongly favorable attitudes were translated into commitment, which is more consistent with an attitude-strength interpretation than with a simple deficit model. Accordingly, the findings are better read as evidence of a bounded vulnerability in intention formation than as evidence that ambivalence is a general barrier to adolescent physical activity. In substantive terms, the findings suggest that favorable attitudes may be necessary but not always sufficient for strong intention formation in adolescent leisure-time PA (Petty et al., 2023).

The basic prospective pathway in the model was consistent with reasoned action and TPB logic. Adolescents who evaluated regular leisure-time MVPA more favorably at baseline reported stronger intentions later, and those intentions in turn predicted subsequent leisure-time PA. The approximately two-week lags are consistent with this proximal interpretation; however, they also mean that the present findings speak most directly to short-term prospective processes rather than to more durable behavioral change. Critically, because the same leisure-time PA outcome was not assessed at baseline, the model predicts later self-reported behavior but does not directly demonstrate behavioral change; the observed prospective association may partly reflect behavioral continuity across the study period. This pattern aligns with longstanding theory and with meta-analytic evidence showing that attitudes are important antecedents of intention and that intention is a key proximal predictor of behavior in health domains and physical activity specifically (Ajzen, 1991; Fishbein & Ajzen, 2010; Hagger et al., 2002; R. R. C. McEachan et al., 2011). It also complements recent adolescent leisure-time PA research that continues to rely on TPB-based or integrated social-cognitive models to explain motivational processes over time (Pasi et al., 2021; Su et al., 2024). Because leisure-time MVPA is relatively discretionary compared with required school activity, it is plausible that adolescents’ own evaluations are especially consequential in this context. At the same time, the present findings indicate that favorable attitudes do not operate with equal strength across adolescents.

The core contribution of the study lies in the moderation findings. Felt ambivalence did not simply lower intention in a uniform way. Rather, it weakened the extent to which favorable attitudes were translated into later intention. That said, this pattern should not be overgeneralized. Because the main effect of ambivalence on intention was non-significant, the present data do not indicate that ambivalence is uniformly detrimental; instead, they suggest that ambivalence matters primarily when adolescents otherwise hold favorable attitudes. This distinction is theoretically important. Priester and Petty (1996) conceptualized felt ambivalence as the subjective experience of mixed reactions, conflict, or indecision, and attitude-strength research has long suggested that less ambivalent attitudes are more predictive of later intentions and behavior than highly ambivalent ones (Armitage & Conner, 2000; Petty et al., 2023). Within TPB research, Conner et al. (2003) likewise showed that attitudinal ambivalence can moderate prospective model relations. The present findings are broadly consistent with that logic, but they do not identify the specific mechanism by which favorable attitudes fail to become intentions. At the same time, the present design cannot determine whether ambivalence itself is the active causal factor or whether it partly indexes unresolved practical barriers, such as time pressure, academic competition, embarrassment, or limited access to activity opportunities. Adolescents may genuinely see regular leisure-time MVPA as beneficial, worthwhile, or enjoyable, yet simultaneously experience it as effortful, inconvenient, embarrassing, or in competition with academics and other leisure activities. Under those conditions, an overall attitude can remain positive while still being less settled and less action-guiding. On balance, the findings are most consistent with a conditional and context-sensitive role for ambivalence, rather than a broad stand-alone explanation of adolescent leisure-time PA.

The moderated mediation findings further clarify where ambivalence matters in the motivational sequence. The indirect effect of attitude on later leisure-time PA through intention remained positive at all examined levels of ambivalence, indicating that favorable attitudes still conferred some motivational advantage even among more conflicted adolescents. At the same time, because Wave 3 leisure-time PA was assessed by self-report, the magnitude of this indirect pathway—as well as the direct attitude–PA association and the explained variance in Wave 3 PA—may be somewhat inflated by recall limitations, response tendencies, social desirability, or shared self-report method variance. These estimates should therefore be interpreted as approximate indicators of association rather than as precise estimates of objective behavioral effects. The moderated indirect effect is therefore best viewed as evidence of attenuation, not reversal: higher ambivalence reduced the size of the indirect pathway, but the pathway remained positive even at high ambivalence. Although this moderation should be interpreted as small in standardized terms (β = −0.082), its practical meaning is clearer when expressed in conditional effects: from low to high ambivalence, the indirect effect declined from 0.268 to 0.194 (about 28%), and the corresponding attitude → intention simple slope declined from approximately 0.67 to 0.49 (about 27%). Even so, these differences are better interpreted as a bounded and practically modest point of vulnerability in intention formation, rather than as evidence of a large downstream behavioral leverage point or a strong stand-alone intervention target. Together with the non-significant main effect of ambivalence on intention and its non-significant direct association with later PA, this pattern suggests that ambivalence operated chiefly by reducing the efficiency with which favorable evaluations were converted into intention. This interpretation remains provisional, however, because the short lags and absence of baseline leisure-time PA leave open the possibility that the model partly reflects short-term consistency in self-reported cognitions and behavior. At the same time, attitude retained a small direct association with Wave 3 leisure-time PA, indicating partial rather than full mediation. This remaining direct effect suggests that favorable attitudes may also shape later activity through pathways not captured by the present mediator, such as greater openness to spontaneous activity opportunities or greater persistence once activity is initiated (Conner et al., 2003; Petty et al., 2023).

The findings for habit also deserve brief consideration. Baseline habit strength positively predicted both subsequent intention and later leisure-time PA. This is consistent with work indicating that repeated physical activity can become relatively automatic, low effort, and less dependent on active deliberation, as well as with meta-analytic evidence that including past behavior attenuates some reasoned-action effects (Hagger, 2019; Hagger et al., 2018). Importantly, however, the ambivalence interaction remained significant after adjusting for habit. This suggests that the central moderation effect is not fully attributable to automaticity or prior behavioral carryover. Rather, even after accounting for a rival process rooted in repeated behavior, felt ambivalence remained associated with variation in the extent to which favorable attitudes related to intention. The covariate findings for gender and grade were secondary, but they suggest that intention formation and leisure-time PA are also embedded in broader developmental and social constraints that were not directly modeled here.

Theoretically, the present study makes several contributions. First, it refines understanding of the motivational sequence in adolescent leisure-time PA. Rather than suggesting that the intention–behavior gap should be replaced, the findings indicate that existing reasoned action/TPB models can be sharpened by considering an earlier source of variation in intention formation: favorable attitudes are not equally action-guiding when adolescents feel conflicted about the behavior. Second, the study offers a parsimonious integration of reasoned action logic with attitude-strength theory by locating felt ambivalence as a first-stage moderator of the attitude–intention pathway. Third, it extends a sparse PA ambivalence literature beyond the limited evidence currently available from adolescents with critical congenital heart disease (Fox et al., 2023) and beyond adolescent TPB-based PA studies that model attitudes mainly in terms of mean level rather than strength or conflict (Pasi et al., 2021; Su et al., 2024), specifically by prospectively testing felt ambivalence as a moderator of the attitude–intention pathway in general adolescents’ leisure-time PA while adjusting for PA habit. Taken together, the contribution is best understood as specifying a boundary condition within established social-cognitive models of adolescent PA, not as proposing a conceptual departure from them. At the same time, the present evidence supports a relatively specific claim: felt ambivalence appears relevant to short-term intention formation in this school-based sample, but it should not yet be regarded as a comprehensive explanation for adolescent leisure-time PA behavior.

These findings also have tentative practical implications. Although the present study did not test an intervention, the pattern observed here suggests that efforts to promote adolescent leisure-time MVPA may not be maximally effective if they focus only on making attitudes more positive. For some adolescents, the key problem may not be the absence of perceived benefits, but the coexistence of favorable views with unresolved concerns about time, fatigue, embarrassment, lack of access, or conflict with academic demands and competing leisure options. This may be especially salient in Shanghai and similar urban Chinese settings, where adolescents often navigate substantial academic pressure, heavy homework demands, and tightly structured school schedules that compress discretionary time for leisure-time activity (Zhu et al., 2017, 2021). In such contexts, ambivalence may reflect not only personal hesitation but also a real conflict between valuing physical activity and managing school-related obligations. Physical activity opportunities are also shaped by school- and community-level supports such as PE provision, access to facilities, and local activity opportunities (Wang et al., 2017; Liu et al., 2023). If future intervention studies support this interpretation, a promising hypothesis for intervention research is that explicitly surfacing and addressing these mixed reactions might be more useful than assuming that positive beliefs alone will yield commitment. Tentatively, this suggests that students who “like the idea” of being active may need support in resolving conflict, not simply more persuasion about benefits. In similar settings, future intervention research could examine approaches such as making activity more feasible within constrained schedules, strengthening accessible after-school opportunities, and aligning activity promotion with school and family priorities rather than treating leisure-time PA as a purely individual choice. At the same time, the present results do not imply that reducing ambivalence alone would produce a large universal change in behavior. Rather, the moderation suggests a testable possibility for future intervention studies: when adolescents already hold favorable attitudes but remain conflicted, strategies that help resolve mixed reactions might modestly strengthen intention formation and make standard benefit-focused messages more effective. Thus, the practical value of the finding is less that ambivalence is a dominant lever and more that it identifies when conventional attitude-based approaches may be less efficient unless paired with conflict-resolution support. This implication is clearest for intention formation; implications for behavioral change should be interpreted more cautiously because the Wave 3 PA outcome was self-reported and baseline leisure-time PA was not measured. The positive role of habit additionally suggests that future intervention studies could examine whether work on ambivalence is usefully paired with repeated, context-stable opportunities for activity that can help emerging intentions become more routinized over time (Fox et al., 2023; Hagger, 2019).

Several limitations should be acknowledged. First, although the design was prospective, it was nonexperimental; therefore, causal conclusions remain limited, and reciprocal or omitted-variable explanations cannot be ruled out. Second, all focal constructs, including the behavioral outcome, were assessed by self-report. Although this is common and often practical in school-based research, physical activity measurement in youth has recognized reliability and validity challenges (Sirard & Pate, 2001). In particular, the Wave 3 leisure-time PA index may be affected by recall error, response tendencies, and social desirability. As a result, associations involving the behavioral outcome, including path coefficients, indirect effects, and the proportion of explained variance in Wave 3 PA, may be somewhat inflated and should not be interpreted as precise estimates of objectively measured activity. Moreover, because the behavioral outcome was operationalized as a brief two-item index, it offers less psychometric depth and less behavioral detail than a more comprehensive or device-based assessment of adolescent PA. In the context of repeated school-based group administration across three waves, we used a brief self-report indicator to keep data collection feasible and respondent burden low while preserving correspondence with the focal attitude and intention measures; however, that practical advantage came at the cost of lower measurement precision than device-based monitoring could provide. The three-wave design provides temporal separation among constructs, but it does not eliminate the limitations inherent in self-reported PA assessment. Future studies would benefit from device-based or more detailed repeated assessments of leisure-time MVPA. Third, a key design limitation is that the same leisure-time PA outcome was not assessed at baseline. Consequently, we could not determine whether the modeled pathways predicted change in leisure-time PA or instead partly reflected behavioral stability across waves. Although Wave 1 PA habit was statistically controlled, this does not substitute for direct adjustment for baseline leisure-time PA. Accordingly, the present findings should be interpreted as prospective prediction of later behavior, not as evidence of behavior change. Future studies should include matched baseline and follow-up assessments of leisure-time MVPA so that change can be modeled more directly. Fourth, the approximately two-week intervals between waves were selected to capture relatively proximal motivational processes, but they may have been too short to reflect more durable behavioral dynamics in adolescent leisure-time PA. As a result, some observed associations may partly reflect short-term reporting consistency or temporary stability in cognitions and behavior, rather than longer-term developmental or behavioral processes. Accordingly, the present findings are best interpreted as evidence of short-term prospective coupling. Future studies should examine whether the same pattern replicates across longer follow-up intervals and with repeated assessments over months rather than weeks. Fifth, students were nested within 10 schools, but the analyses were conducted at the individual level and did not explicitly model school-level clustering. Because responses from students within the same school may be correlated, standard errors may be somewhat anti-conservative and statistical significance may therefore be somewhat overstated. The relatively small number of schools also limits strong inferences about between-school heterogeneity. Future studies should account for clustering more directly, for example through cluster-adjusted or multilevel SEM approaches when a larger number of schools is available. Sixth, participants were recruited from schools in Shanghai, so generalization to adolescents in other regions or settings should be made cautiously. This is not only a geographic limitation but also a contextual one. Shanghai adolescents are embedded in an urban, academically intensive environment in which homework burden, examination pressure, and tightly structured school schedules may shape leisure-time physical activity opportunities and the kinds of conflict captured by felt ambivalence (Zhu et al., 2017, 2021). Moreover, school- and community-level supports for physical activity vary across settings (Wang et al., 2017; Liu et al., 2023). These contextual influences were not directly measured in the present study, so they should be viewed as plausible boundary conditions rather than tested explanations. Accordingly, the present pattern may be especially relevant to similar high-pressure urban school contexts and should not be assumed to generalize unchanged to adolescents in rural areas, other Chinese regions, or countries with different educational systems, family expectations, and leisure-time opportunities. Finally, observed composites were used for parsimony, but future research could test latent-variable interactions, examine longer follow-up periods, and compare felt ambivalence with indirect ambivalence indices derived from separate positive and negative evaluations (Priester & Petty, 1996). Experimental work would be especially valuable for testing whether reducing ambivalence strengthens intention formation for regular leisure-time MVPA (Armitage & Conner, 2000).

## 5. Conclusions

The present findings suggest that adolescents can hold genuinely favorable views of regular leisure-time MVPA while remaining sufficiently conflicted for those views to translate fully into intention. These results refine the existing adolescent PA models by indicating that felt ambivalence may function as a boundary condition on the attitude–intention link. More specifically, the study provides prospective support for a theoretically relevant moderation pattern consistent with an attitude-strength account of intention formation. However, the present nonexperimental design does not establish felt ambivalence as a key explanatory mechanism or identify the specific process by which favorable attitudes fail to translate into stronger intention. The clearest implication of the study concerns intention formation; implications for later leisure-time PA should be interpreted more cautiously because the behavioral outcome was self-reported and baseline leisure-time PA was not measured. Future research should test whether this moderation pattern replicates over longer intervals and under designs better suited to causal explanation.

## Figures and Tables

**Table 1:** Descriptive Statistics, Internal Consistency, and Zero-Order Correlations Among Study Variables.

Variable	M	SD	α	1	2	3	4	5	6
1. W1 PA attitude	6.21	1.00	0.95	—					
2. W1 felt ambivalence	2.89	1.56	0.91	−0.28	—				
3. W1 PA habit	4.42	1.64	0.93	0.50	−0.24	—			
4. W2 PA intention	4.62	1.20	0.94	0.62	−0.24	0.55	—		
5. W3 leisure-time PA	3.62	1.25	—	0.42	−0.13	0.45	0.55	—	
6. Grade	8.70	1.74	—	−0.02	0.02	−0.05	−0.05	−0.10	—
7. Gender	0.50	0.50	—	−0.14	−0.04	−0.16	−0.17	−0.21	0.09

All statistics are based on the complete-case analytic sample (N = 1714). Cronbach’s α is reported for the multi-item psychometric scales only and is not shown for the Wave 3 leisure-time PA index or demographic covariates. The Wave 3 leisure-time PA variable was treated as a brief behavioral indicator rather than a psychometric scale and should therefore be interpreted as a brief indicator of leisure-time MVPA frequency and regularity rather than as a comprehensive assessment of adolescent PA. Correlations are presented below the diagonal. Higher scores indicate more favorable physical activity attitudes, greater felt ambivalence, stronger PA habit, stronger PA intention, and higher leisure-time PA. Gender was coded as 0 = male and 1 = female. W1 = Wave 1; W2 = Wave 2; W3 = Wave 3.

**Table 2:** Parameter Estimates for the Moderated Mediation Model Predicting Wave 3 Leisure-Time Physical Activity.

Parameter	b	SE	z	*p*	β	95% CI
Panel A. Structural paths						
W2 PA intention ← W1 PA attitude	0.577	0.031	18.54	<0.001	0.483	[0.517, 0.638]
W2 PA intention ← W1 felt ambivalence	−0.024	0.016	−1.49	0.137	−0.031	[−0.055, 0.008]
W2 PA intention ← W1 attitude × W1 ambivalence	−0.058	0.017	−3.41	<0.001	−0.082	[−0.095, −0.028]
W2 PA intention ← W1 PA habit	0.215	0.018	11.65	<0.001	0.295	[0.180, 0.253]
W2 PA intention ← Gender	−0.127	0.044	−2.92	0.004	−0.053	[−0.212, −0.044]
W2 PA intention ← Grade	−0.012	0.012	−1.01	0.313	−0.018	[−0.036, 0.012]
W3 leisure-time PA ← W2 PA intention	0.400	0.031	12.77	<0.001	0.385	[0.338, 0.461]
W3 leisure-time PA ← W1 PA attitude	0.100	0.034	2.93	0.003	0.080	[0.032, 0.165]
W3 leisure-time PA ← W1 felt ambivalence	0.018	0.018	1.00	0.318	0.022	[−0.018, 0.052]
W3 leisure-time PA ← W1 PA habit	0.139	0.022	6.29	<0.001	0.183	[0.095, 0.184]
W3 leisure-time PA ← Gender	−0.245	0.050	−4.87	<0.001	−0.098	[−0.343, −0.147]
W3 leisure-time PA ← Grade	−0.043	0.014	−3.02	0.003	−0.059	[−0.071, −0.015]
Panel B. Conditional indirect effects of W1 PA attitude on W3 leisure-time PA through W2 PA intention						
Low ambivalence (−1 SD)	0.268	0.029				[0.215, 0.328]
Mean ambivalence	0.231	0.023				[0.188, 0.277]
High ambivalence (+1 SD)	0.194	0.021				[0.154, 0.236]
Index of moderated mediation	−0.023	0.007				[−0.039, −0.011]
Panel C. Model fit and explained variance						
χ^2^, *p*	0.001, 0.972					
CFI	1.000					
RMSEA	0.000					
SRMR	<0.001					
R^2^ for W2 PA intention	0.468					
R^2^ for W3 leisure-time PA	0.350					

Note. N = 1714. b = unstandardized coefficient; β = standardized coefficient; CI = 95% bootstrap confidence interval; CFI = Comparative Fit Index; RMSEA = root mean square error of approximation; SRMR = standardized root mean square residual; PA = physical activity. W1 PA attitude and W1 felt ambivalence were mean-centered before forming the interaction term. Indirect effects and the index of moderated mediation were estimated with 5000 bootstrap resamples. Because the final model had only one degree of freedom, the global fit indices are reported for completeness and should not be overinterpreted. Low and high ambivalence correspond to −1 SD and +1 SD from the mean, respectively. Covariances, intercepts, and residual variances from the full lavaan output are omitted for brevity. Gender was coded as 0 = male and 1 = female. W1 = Wave 1; W2 = Wave 2; W3 = Wave 3.

## Data Availability

The data supporting the findings of this study are not publicly available because of school-level agreements and participant privacy protections but are available from the corresponding author upon reasonable request.
